# High case fatality cholera outbreak in Western Kenya, August 2010

**DOI:** 10.11604/pamj.2013.15.109.2270

**Published:** 2013-07-24

**Authors:** Dickens Onyango, Shirley Karambu, Ahmed Abade, Samuel Amwayi, Jared Omolo

**Affiliations:** 1 Ministry of Public Health and Sanitation, Kenya; 2 Field epidemiology and laboratory management training program (FELTP), Kenya

**Keywords:** Outbreak, Kuria West, cholera

## Abstract

**Introduction:**

Cholera is a disease caused by the bacterium *Vibrio cholera* and has been an important public health problem since its first pandemic in 1817. Kenya has had numerous outbreaks of cholera ever since it was first detected there during 1971. In August 2010 an outbreak of cholera occurred in Kuria West District spreading to the neighboring Migori District. We conducted an investigation in order to determine the magnitude of the problem and institute control measures.

**Methods:**

In order to update the line lists we reviewed records in Migori and Kuria district hospitals and conducted active case search in the community between 30th August and 6th September 2010. Data was analyzed using Epi-Info 3.5.2.

**Results:**

A total of 114 cases and with 10 deaths (Case Fatality Rate = 9%) were documented. The index case was an 80 years old woman from Mabera Division who had hosted a cultural marriage ceremony a day before the outbreak. The mean age of case patients was 34.5 years (Standard Deviation=23.4) with a range 5 to 80 years. Females accounted for 61.4% of cases; people aged 10-39 years accounted 46.9%, those 40-69 years accounted for 29.2% and those above 70 years accounted for 9.7% of the cases. Sixty percent of deaths occurred among patients aged 50 years and over, case fatality rate was highest in this age group (16.7%) followed by those aged 40-49 years (12.5%), 20-29 years (10%) and 10-19 years (4.8%). The outbreak was confirmed within 2 weeks of onset after one (16.7%) of the six samples taken tested positive for *V. cholera* (serotype Inaba).

**Conclusion:**

High case fatality rate and late laboratory confirmation was noted in this outbreak. There was urgent need to capacity build the districts on cholera case management, outbreak management, and equip the Migori District Hospital laboratory to allow prompt confirmation.

## Introduction

Cholera is still an important public health problem globally. There have been several large outbreaks of the disease across the world [1, 2]. About 2.8 million cases of cholera occur annually, the incidence is 2.0 cases per 1000 people at risk. Countries in southern Asia and Africa have the highest incidences [3]. Approximately 66.0% of global cholera cases and 87.6% of fatalities occur in Sub-Saharan Africa. It kills an estimated 91,000 annually, the mortality rate varies from 0.1 per 100,000 in developed countries to 15.2 per 100,000 in Africa [3]. Kenya experienced a resurgence of cholera outbreaks in the period from 2008 to 2010 [4]. Although these outbreaks occurred in many parts of the country, the Western region was more affected [5].

The World Health Organization recommends that cholera case fatality rates should not exceed one percent if cases are properly treated [6]. However, cholera outbreaks have continued to kill greater than one percent of the cases. In 2010, for instance, 46% of countries which reported cholera outbreaks had case fatality rates exceeding the World Health Organization recommended level [6]. Cholera outbreaks have occurred in Kenya since 1971, the average case fatality rate in these epidemics was 3.57% [7]. There were 11,769 cases and 274 deaths (case fatality rate 2.33%) in 2009 and 3354 cases and 72 deaths (case fatality rate 2.1%) in 2010 [7, 8].

In august 2010, an outbreak of acute watery diarrhea occurred in Kuria West District in the western part of Kenya and spilled over to neighboring Migori District. The Ministry of Public Health and Sanitation mandated the Kenya Field Epidemiology Training Program to investigate and assist with controlling the outbreak. The objective of our investigation was to determine the magnitude of the outbreak, characterize the outbreak and assess the district's preparedness to deal with the outbreak.

## Methods

**Study Site:** Kuria West District is one of the thirty-eight districts that form Nyanza Province in western Kenya. It borders Migori District to the North, Trans-mara and Kuria East Districts to the East and the Republic of Tanzania to the South. The district is located between latitudes 00150 and 00300 south of the Equator and longitudes 34015′ and 34030′ east of the Prime Meridian. The total area covered by the district is 407.9 square kilometers. The district is divided into three administrative divisions namely Kehancha, Masaba and Mabera.

**Study Design:** The investigation was carried out from 30^th^ August 2010 to 6^th^ September 2010 at Migori and Kuria West Districts. We conducted a retrospective review of outpatient and inpatient records in all health facilities in the district. We further obtained line lists from the two districts and harmonized them. Active case search was conducted in the community. Data obtained from the line lists were analyzed using Epi Info version 3.5.2 and Ms Excel 2007.

To assess the district's level of preparedness, we conducted key informant interviews with members of the district health management team (DHMT). They were asked open-ended questions on outbreak response, the performance of their surveillance system, the laboratory response and clinical management of patients.

**Case definitions:** A suspected case was any resident of Kuria West or Migori District, aged 2 years or more, presenting with profuse effortless watery diarrhea; more than three motions in 24 hours of sudden onset with or without vomiting on or after 1^st^ August 2010. A confirmed case was any resident of the two districts with a compatible clinical presentation and with *Vibrio cholera* confirmed by culture or rapid test.

## Results

**Figure 1:**
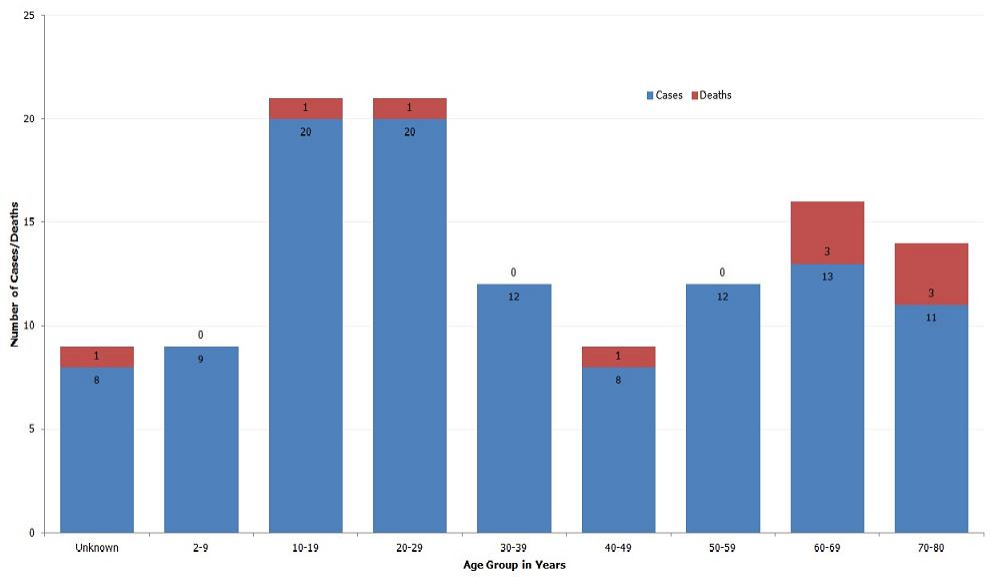
Distribution of suspected cholera cases and deaths by age group, Kuria West district, 2010

One hundred and fourteen met the criteria for suspected cases and one case, 16.7% of the six tested specimens, was confirmed to have *Vibrio cholera, inaba* by culture. Sixty-one percent (n = 70) of cases were females. The mean age of cases was 34.5 years, (standard deviation = 23.4). Ten deaths (case fatality rate = 9%) occurred during this outbreak. Females accounted for 60% (n= 6) of deaths. The mean age of the dead was 57.2 years (standard deviation= 22). Most cases (36%, n= 41) were aged between 10 to 29 years whereas most deaths (60%, n= 6) occurred in the age group 60 to 80 years. Figure 1 shows the distribution of cases and deaths according to age group.

**Figure 2:**
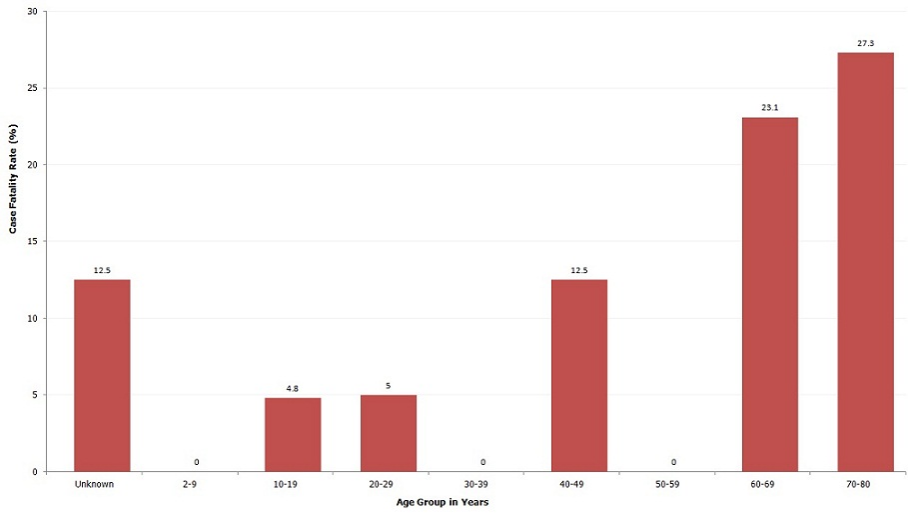
Case fatality rate from suspected cholera by age group, Kuria West district, 2010

Those aged 70 to 80 years had the highest case fatality rate, 27.3%. Figure 2 shows the distribution of case fatality rate by age group in years. Two of the suspected cases were from Migori District; Mabera Division had the highest attack rate 0.3%. Table 1 below presents the distribution of cases by administrative regions.

**Table 1:** Distribution of cholera cases by administrative region

Division	No of Cases	Percentage	Population	Attack Rate
Mabera	96	86	33,113	0.3%
Masaba	9	8	26,957	0.03%
Isbania	4	3	23, 476	0.0012%
Kehancha	1	1	93,039	0.0012%
Migori	2	2	355, 562	0.0001%
**Total**	114	100	532, 147	0.021

### Epidemic curve

**Figure 3:**
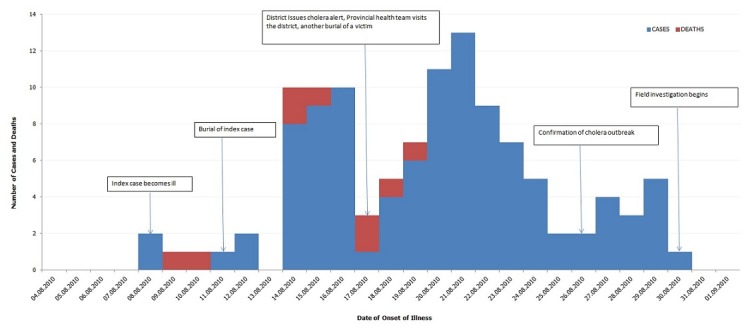
Distribution of suspected cholera cases and deaths by date of onset of illness (Epidemic curve), Kuria West district, 2010

The index case fell sick on 8th August and died on 9^th^ August, this case came from Kuria West who sought care and died in a private facility in Migori District. The first case admitted in Kuria West was an 80-year-old female who fell ill on 11 August and died on 12th at a health centre in the district. There was a rapid rise in the number of cases reported following the burial of this case, with a peak occurring on 21st August 2010 followed by a steady decline in the number of cases reported. This first case in Kuria West is linked to a traditional marriage ceremony in the village where mostly elderly people feasted prior to the onset of cases. The elderly woman who hosted the ceremony subsequently fell ill and died. Other than the peak on 21^st^ there were two others that occurred following burial ceremonies. Figure 3 presents the epidemic curve.

### District preparedness

The district was ill prepared to handle this outbreak. The DHMT were not trained on integrated disease surveillance and response. They lacked skills on how to manage an outbreak. The surveillance system was weak; it had failed to detect the outbreak in a timely manner. Line listing of the initial cases was done retrospectively. Laboratory response during this outbreak was feeble, the district laboratory lacked capacity to detect *Vibrio cholera* and most health workers could not competently take a rectal swab. There was a delay in taking specimen; the first specimen was taken 10 days after the onset of the outbreak. The specimen had to be transported more than 300 kilometres away for testing. There was a shortage of oral rehydration and intravenous fluids at the time of the outbreak. Cholera treatment centres were not set up and cases were referred to facilities in the neighbouring Migori District.

## Discussion

This was an outbreak of acute watery diarrhea; one case was confirmed to be cholera with the serotype being inaba. The serotype isolated is the most common cause of cholera epidemics in Kenya and other parts of Africa [9]. Other organisms like enterotoxigenic E. coli that can cause similar outbreaks [4, 10], these were not ruled out due to poor laboratory capacity. Although there was only one confirmed case and other organisms were not ruled out, this outbreak was likely to be due to cholera. In addition to the one confirmed case, there were two other cases epidemiologically linked to the confirmed. The number of rectal swabs taken was low compared to the number of cases because of lack of transport media, inadequate expertise and negative attitude of the community toward rectal swabbing. Delay in transporting the specimen and other factors surrounding the handling of specimen could have compromised laboratory results.

Cholera has remained an important cause of epidemics in Western Kenya. Drinking water from the lake or streams and feasting in funerals have been identified as significant risk factors for cholera outbreaks [11]. This outbreak followed a traditional marriage ceremony and spikes in cases occurred following funerals. This is consistent with an outbreak in Guinea Bissau where the attack rate during the week following funerals was higher in villages where bodies were not disinfected [12]. Eating at a funeral with non-disinfected corpse is a recognized risk factor for cholera [12]. The burial of cholera victims without disinfection and feasting at these funerals definitely fuelled the spike in cases. Case fatality rate during this outbreak was higher than the World Health Organization recommendation. This fatality rate was much higher than Kenya's case fatality rate for cholera outbreaks in 2010 [8]. Correct case management by qualified staff, availability of rehydration fluids and good coordination are associated with low case fatality rate [13]. The high case fatality rate in this outbreak may have been due to poor case management, inadequate skills among health staff, and weaknesses in the surveillance system and poor management support. A delay in confirming the outbreak and instituting appropriate measures contributed immensely to the many preventable deaths that occurred. This is consistent with the findings of the 2008 cholera outbreak in Zimbabwe; inappropriate cholera case management with inadequate use of oral rehydration therapy, inappropriate use of antibiotics, and a shortage of experienced healthcare professionals contributed to high case fatality [1]. A break down in health service delivery was also blamed for the high case fatality rate in the Zimbabwe outbreak [14]. Poor access to health facilities and lack of knowledge among rural folk has been blamed for high case fatality rate in other outbreaks [15]. In an outbreak in Guinea Bissau, those who died were six times more likely not to have sought care in a health center [16].

This investigation has one important limitation. An analytical study to determine risk factors for the outbreak was not done due to the late timing of the investigation.

## Conclusion

This was a point source outbreak, initial exposure occurred during a marriage ceremony with subsequent transmissions during funerals of deceased case patients. The case fatality rate was higher than the World Health Organization recommendation. The surveillance system in the district was weak, it was unable to detect the outbreak in a timely manner and several weaknesses were noted in the line listing of cases. The district lacked laboratory capacity to confirm the outbreak and delayed in responding to the outbreak.

## References

[1] Ahmed S, Bardhan PK, Iqbal A, Mazumder RN, Khan AI (2011). The 2008 cholera epidemic in Zimbabwe: experience of the icddr,b team in the field. J Health Popul Nutr..

[2] Outbreak news (2008). Severe acute watery diarrhoea with cases positive for Vibrio cholerae, Viet Nam. Wkly Epidemiol Rec..

[3] Ali M, Lopez AL, You YA, Kim YE, Sah B, Maskery B, Clemens J (2012). The global burden of cholera. Bull World Health Organ.

[4] Saidi SM, Yamasaki S, Lijima Y, Kariuki S (2011). Cholera-like diarrhoea due to Salmonella infection. J Infect Dev Ctries..

[5] Feikin DR, Tabu CW, Gichuki J (2010). Does water hyacinth on East African lakes promote cholera outbreaks?. Am J Trop Med Hyg..

[6] WHO: Global Health Observatory (GHO) (2010). Reported cholera case fatality rate; Situation and trends.

[7] WHO: Cholera Country Profile: Kenya

[8] (2010). Cholera Situation in Kenya, 2010.

[9] Smith AM, Keddy KH, De Wee L (2008). Characterization of cholera outbreak isolates from Namibia, December 2006 to February 2007. Epidemiol Infect.

[10] Harris AM, Chowdhury F, Begum YA, Khan AI (2008). Shifting prevalence of major diarrheal pathogens in patients seeking hospital care during floods in 1998,2004, and 2007 in Dhaka, Bangladesh. Am J Trop Med Hyg..

[11] Shapiro RL, Otieno MR, Adcock PM (1999). Transmission of epidemic Vibrio cholerae O1 in rural western Kenya associated with drinking water from Lake Victoria: an environmental reservoir for cholera?. Am J Trop Med Hyg..

[12] Gunnlaugsson G, Einarsdottir J, Angulo FJ (1998). Funerals during the 1994 cholera epidemic in Guinea-Bissau, West Africa: the need for disinfection of bodies of persons dying of cholera. Epidemiol Infect..

[13] Gbary AR, Sossou RA, Dossou JP, Mongbo V, Massougbodji A (2011). The determinants of the low case fatality rate of the cholera epidemic in the Littoral department of Benin in 2008. Sante Publique.

[14] Mason PR (2009). Zimbabwe experiences the worst epidemic of cholera in Africa. J Infect Dev Ctries..

[15] Quick RE, Vargas R, Moreno D, Mujica O, Beingolea L (1993). Epidemic cholera in the Amazon: the challenge of preventing death. Am J Trop Med Hyg..

[16] Gunnlaugsson G, Angulo FJ, Einarsdottir J, Passa A (2000). Epidemic cholera in Guinea-Bissau: the challenge of preventing deaths in rural West Africa. Int J Infect Dis..

